# *Aeromonas hydrophila* Infection following Leech Therapy for the Treatment of Nipple–Areola Complex Congestion after Breast Reduction: A Case Report

**DOI:** 10.1055/s-0043-1776696

**Published:** 2024-04-04

**Authors:** Matteo Torresetti, Benedetta Peltristo, Francesco Mauro Junior Taddei, Giovanni Di Benedetto

**Affiliations:** 1 Department of Experimental and Clinical Medicine, Clinic of Plastic and Reconstructive Surgery, Marche Polytechnic University Medical School, Ancona, Italy

**Keywords:** leech, infection, nipple–areola complex, venous congestion, reduction mammaplasty

## Abstract

Several strategies for the management of venous congestion of the nipple–areola complex (NAC) after reduction mammaplasty have been proposed. Among these, hirudotherapy represents an ancient but still effective method, even though the risk of infections related to leeches should be considered. We report a peculiar case of breast infection and sepsis after leech therapy in a patient who underwent a reduction mammaplasty. A prompt surgical debridement of the wounds and necrotic tissues associated with targeted antibiotic therapy led to a fast improvement of clinical conditions, and partial preservation of the NAC was obtained. Accurate knowledge of the clinical presentation of soft tissue infections related to leeching allows for an early diagnosis and would serve as a warning for surgeons who approach such breast cosmetic procedures.

## Introduction

Nipple–areola complex (NAC) necrosis secondary to reduction mammaplasty/mastopexy represents a serious complication with a massive impact on patient satisfaction and well-being. Complete or partial NAC necrosis has been reported in 2 to 6% of breast reductions, respectively, and more frequently in patients with risk factors such as smoking, diabetes, and obesity. The most common cause is inadequate venous drainage with the typical blueish color of the NAC, dark blood at the pinprick, and increased edema. 1 2

Historically, leeches have been used for a variety of medical conditions, with a specific application in Plastic and Reconstructive Surgery on venous congestions since the 1980s. In case of mild venous drainage impairment, leeches can be applied directly to the affected area; the number of leeches and the frequency of treatment are quite variable. Despite the uncontested benefits of hirudotherapy, several complications have been described such as anemia, localized infection, and sepsis, with a reported incidence of infection ranging from 2 to 20%. 3

We present a single case of breast infection related to leech therapy in a patient with NAC venous congestion after a reduction mammaplasty.

## Case

**Fig. 1:**
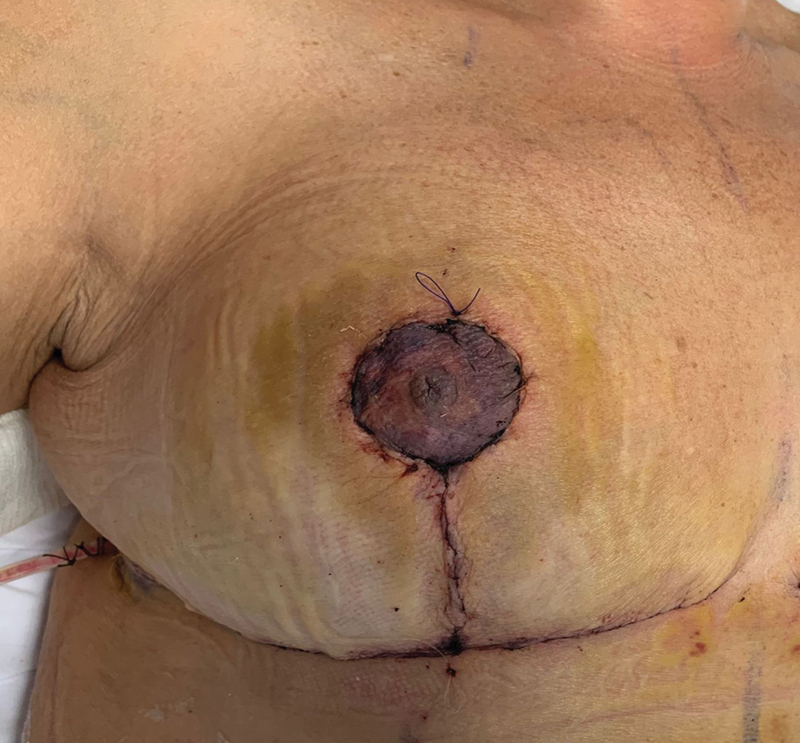
Venous congestion of the nipple–areola complex following reduction mammaplasty.

**Fig. 2:**
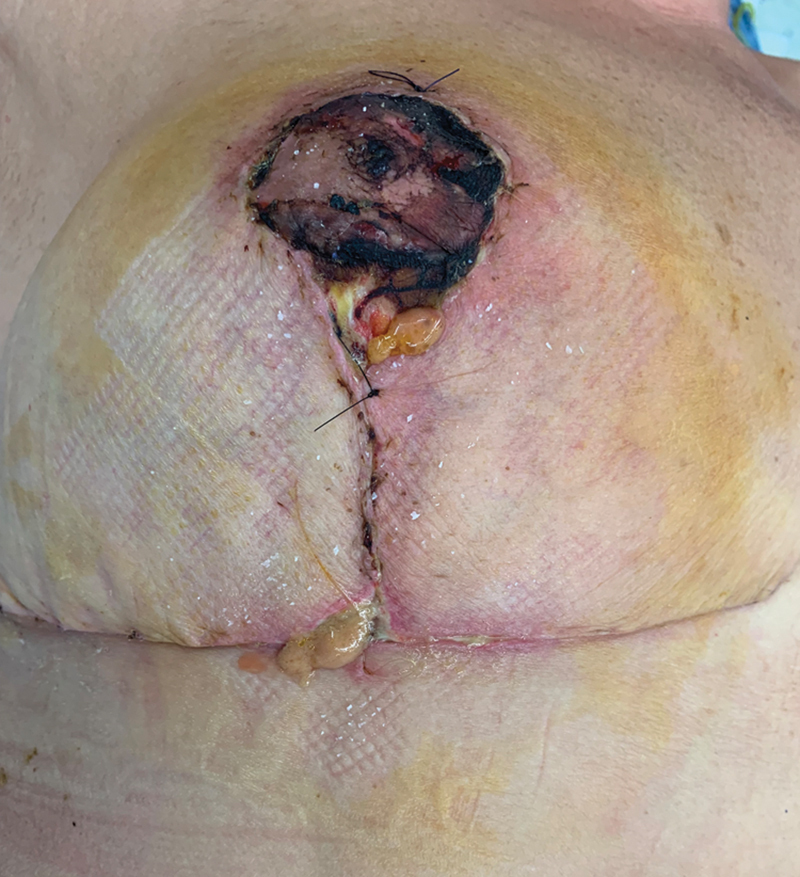
Breast infection after leech therapy on day 8 postoperatively, with wound dehiscence and pus discharge from the wounds.

**Fig. 3:**
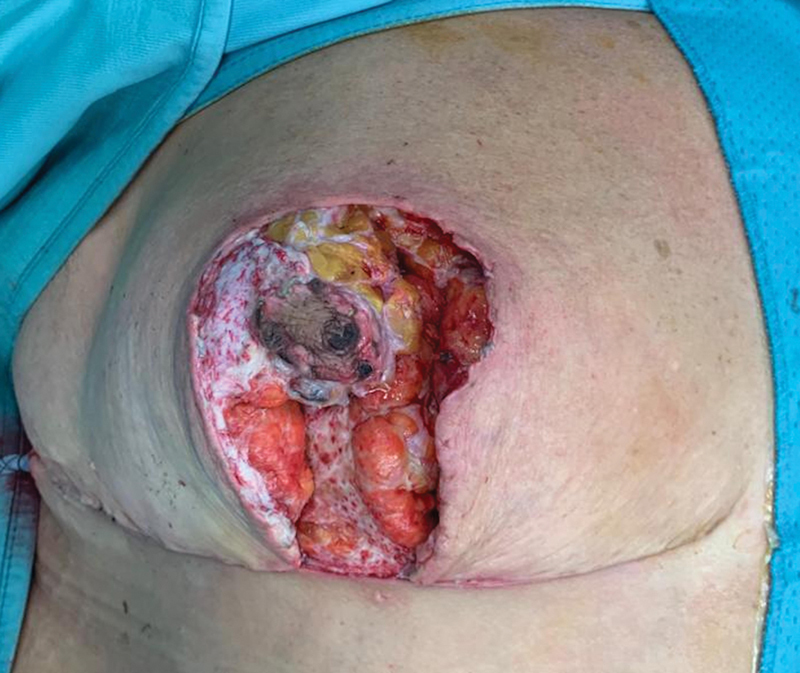
Intraoperative picture showing the surgical debridement of the necrotic tissues and abscess drainage.

**Fig. 4:**
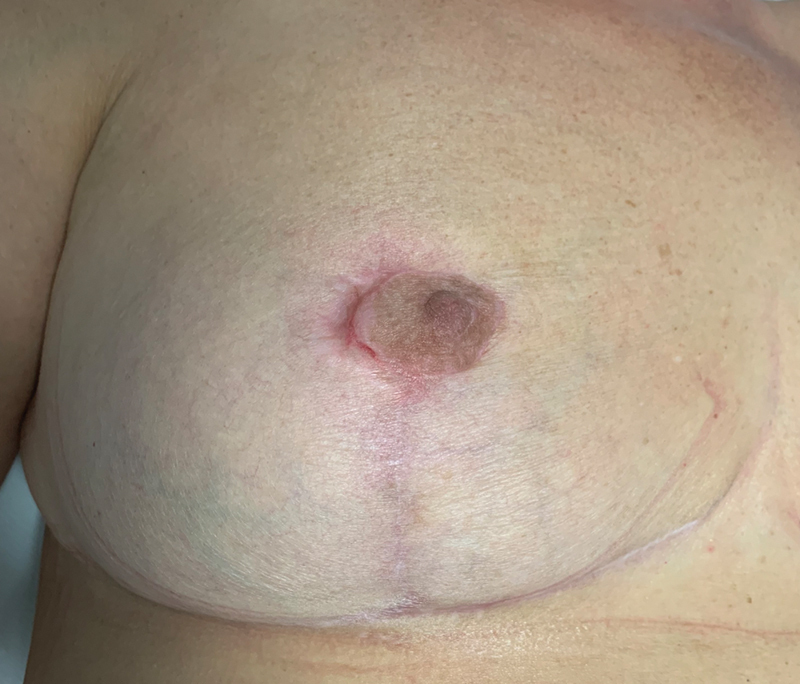
Six-month follow-up visit showing complete healing and partial preservation of the nipple–areola complex.

A 45-year-old woman candidate for body-contouring procedures after massive weight loss was admitted to our department with large and ptotic breasts. The patient underwent bilateral Wise-Pattern Reduction Mammaplasty using a superolateral pedicle and an inferior dermoglandular Ribeiro flap for autoaugmentation. Standard preoperative antibiotic prophylaxis with cefazolin (2 g) was used. On the first postoperative day, the right NAC displayed venous congestion which was managed conservatively with multiple leech applications ( Fig. 1 ). Leech therapy was continued until a visible improvement in NAC perfusion was obtained, and the patient was discharged 5 days postoperatively. On the eighth postoperative day, the patient was readmitted to our hospital for fever (40°C) and clinical presentation of septicemia. Physical examination showed swelling, tenderness, redness, and pus discharge from the wounds on the right breast ( Fig. 2 ). Blood test findings revealed leukocytosis (white blood cells: 21.7 × 10 ^9^ /L) and high levels of C-Reactive Protein (8.8 mg/dL). Surgical treatment consisted of abscess drainage, wound irrigations, and surgical debridement of the necrotic tissues resulting from the colliquated abscess, and reshaping of the remaining breast parenchyma ( Fig. 3 ). *Aeromonas veronii* was isolated in the wound cultures, and intravenous antibiotic therapy was started including meropenem (3 g/d) for 4 days, followed by oral ciprofloxacin (1 g/d) for 8 days. The patient was then discharged 11 days postoperatively and complete wound healing was achieved after 1 month. A 6-month follow-up visit showed an overall satisfactory shape of the breast, and a largely preserved NAC ( Fig. 4 ).

## Discussion

Partial or full-thickness necrosis of the NAC represents a critical complication in terms of aesthetic and functional results after a reduction mammaplasty. Rather than arterial insufficiency, the more common cause is related to venous congestion. In fact, at the basis of the NAC loss, there could be an extremely tight inset of the reshaped breast with a consequent constriction of the pedicle, inadequate preservation of the venous drainage, and/or hematoma. Once detected, the treatment options for NAC congestion are various: from removing any tension created during closure or delayed inset of the pedicle (if torsion is the causal factor) to the more recent use of vacuum-assisted closure therapy, to the “ancient” application of leeches. 4 Each of these methods has pros and cons. The removal of periareolar sutures with delayed wound closure, as well as the delayed inset of the pedicle, is a safe and reliable alternative for the management of NAC congestion. Nevertheless, leaving the wound open may represent a theoretical risk of wound infection or pathological scarring with consequent poor cosmetic outcomes. Moreover, it requires more outpatient visits and an adequate patient compliance.

Further resection of breast parenchyma or resection of deepithelialized keyhole platform represents another alternative by reducing the tension which causes venous congestion. However, a loss of nipple or breast projection could occur.

The negative pressure wound therapy is a simple and reliable option in cases of suffering venous NAC. It demonstrated to reduce significantly perilesional tissue edema. Nevertheless, it may not always be easily accessible and it is a relatively expensive device.

Hirudotherapy for treatment of NAC congestion has been widely reported in the literature. 5 6 7 However, several cases of infection related to leech therapy have been reported, and *Aeromonas* spp. has the most participation in infections. Clinical manifestations usually start in the following 10 days after leeching, even though the timing is quite variable. 8 Our patient started manifesting signs and symptoms of infection 8 days after leech therapy. A prompt diagnosis of infection is essential to avoid more potentially severe complications. In our experience, in addition to conventional practice consisting of local bacteriological samples and broad-spectrum antibiotic, an extensive and early surgical debridement of all necrotic and infected tissues lead to a dramatic improvement in clinical conditions. The fever ceased on the first postoperative day and the patient remained apyretic even after discontinuation of targeted antibiotic therapy, and a fast improvement of blood tests was observed.

Antibiotic prophylaxis is routinely recommended during medical leech therapy in order to reduce the risk of *Aeromonas* infection, although many units did not use prophylaxis or used inappropriate agents until a few years ago. 8 The most commonly used antibiotics for prophylaxis are fluoroquinolones, trimethoprim–sulfamethoxazole, and third-generation cephalosporins, even though emerging multidrug resistance has been reported. Therefore, algorithms for the prevention and control of *Aeromonas* infections associated with hirudotherapy should be further implemented. Among these, new leech culture protocols 9 or regular environmental surveillance culture of leech water have been proposed, even though a standardized practice has not yet been established. 10

The present case would serve as an alert for the possibility of infection transmission when using leech therapy for NAC salvage even in those patients undergoing aesthetic procedures. To date, leech-related breast infections have only been reported in cases of postmastectomy autologous breast reconstruction. 11 Nevertheless, it is known that patients with a history of breast cancer surgery are more prone to surgical site infection due to chemotherapy and/or radiotherapy to which the tissues are subjected. 12 Moreover, it has been observed that oncologic and immunocompromised patients can have severe infections due to *Aeromonas* . 13

The aim of our paper was to report a new case of *Aeromonas* breast infection in a healthy patient with no risk factors who underwent a cosmetic surgery procedure. In this regard, we discourage the use of hirudotherapy in cases where breast implants are used (e.g. augmentation mastopexy with implants) due to the potentially devastating consequences of a soft tissues infection with breast implant involvement.

If on one hand hirudotherapy represents a valid alternative for the management of NAC venous congestion; on the other hand, the potential of soft tissue infections should be carefully considered. When using hirudotherapy for the management of venous congestion after cosmetic breast surgery procedures, both the surgeon and the patients should be aware of these potential complications, and their use should be adequately weighted.
